# Effect of Prosthetic Rehabilitation on Airway Space in Edentulous Patients with Obstructive Sleep Apnea- a Preliminary Observational Study

**DOI:** 10.30476/dentjods.2022.95716.1886

**Published:** 2023-12-01

**Authors:** Preetha Krishnamurthy, Uma Maheswari, Kasim Mohamed

**Affiliations:** 1 Past- Postgraduate, Dept. of Prosthodontics and Crown & Bridge, Sri Ramchandra Institute of Higher Education and Research, Sriher, India; 2 Dept. of Prosthodontics and Crown & Bridge, Sri Ramchandra Institute of Higher Education & Research, Sriher, India

**Keywords:** Complete Denture, Completely Edentulous, Implant-Supported Denture, Obstructive Sleep Apnea, Questionnaire

## Abstract

**Statement of the Problem:**

The common causes of obstructive sleep apnea (OSA) are identified as anatomic and/or functional abnormality in the oral cavity, oropharynx, velopharynx, and hypopharynx leading to compromised airway space and increased collapsibility.

**Purpose:**

This study was conducted to evaluate the effect of implant-supported mandibular complete denture in improving the airway space among completely edentulous patients with OSA and compare it with conventional complete denture.

**Materials and Method:**

In this observational study, completely edentulous individuals were screened with snoring, tiredness, observed apnea, high blood pressure, body mass index, age, neck circumference, and gender (STOP-Bang) questionnaire to evaluate the incidence of OSA. Ten mild-moderate patients were included as study participants. Lateral cephalograms (L1) made at the edentulous state was considered baseline. They were rehabilitated with complete denture prosthesis. One week after denture insertion, two implants were placed in the edentulous mandibular arch. Delayed loading protocol was followed. Lateral cephalogram (L2) was made 6 months after complete denture insertion and 6 months after implant-supported prosthesis (L3). Cephalometric tracings were used to evaluate change in upper airway space (UAS), middle airway space (MAS), and lower airway space (LAS). Repeated measures ANOVA was used to evaluate statistical significance in the airway measurements made at the three intervals. Post hoc Tukey HSD and Bonferroni test were used to assess if the differences obtained were truly significant.

**Results:**

Statistical analysis revealed significant differences in UAS, MAS and LAS between L1, L2 and L3 (*p*< 0.05). Post hoc Tukey HSD indicated that UAS increased significantly at all three intervals followed by LAS and MAS respectively (α=.05). Post hoc Bon-ferroni test indicated that implant-supported mandibular complete dentures had a significant improvement in airway space when compared to conventional complete dentures (α=.05).

**Conclusion:**

Implant-supported mandibular complete denture could be effective in edentulous patients with mild-moderate OSA.

## Introduction

The common causes of obstructive sleep apnea (OSA) are identified as anatomic and/or functional abnormality in the oral cavity, oropharynx, velopharynx, and hypopharynx leading to compromised airway space and increased collapsibility [ 1 ]. Several studies have proved that edentulism can be considered as one of the exacerbating factors that could worsen OSA [ 1 - 2 ]. Complete edentulism causes changes in the postural position of mandible, muscle tone, tongue posture and vertical dimension, thereby affecting airway dimensions. These morphological changes cause challenges in treating such edentulous sleep apneic patients [ 2 - 4 ]; however, it is underdiagnosed and not characterized in elderly edentulous. Diagnosing and treating OSA is important to improve quality of life, reduce morbidity, and mortality [ 5 ]. Alternatives to the standard method of using polysomnography to diagnose OSA are becoming increasingly popular due to its expense and/or limited availability [ 6 ]. Questionnaires are appropriate tools for quick chair side prediction [ 7 ]. Though computed tomography and magnetic resonance imaging have been regarded as the best method of anatomic evaluation, they require more technical expertise, and expenses. In this regard, cephalometric analysis can play a significant role in identifying patients at risk for OSA [ 8 ]. Numerous cephalometric parameters have been associated with its occurrence and it is a simple, safe, and economic method to depict the relevant anatomy of the airway spaces [ 8 ]. Oral appliances like mandibular repositioning device and tongue retainers gained recognition in treating dentulous sleep apneic patients. However, in edentulous patients the retention is not enough to accommodate the appliances intraorally [ 9 ]. Complete dentures have been used in edentulous sleep apneic patients to increase the airway space and reduce apneic episodes [ 6 ]. Owing to short follow up, the effects of wearing dentures is inconclusive [ 10 ]. Moreover, a lack of stability and retention together with residual bone resorption would decrease the chewing ability in patients wearing conventional complete dentures. Implant-supported dentures provide greater retention, stability, and quality of life for edentulous patients [ 11 ]. Considering the benefits conferred by implant-supported complete dentures, the present study aimed to evaluate the impact of implant-supported mandibular complete denture in improving airway spaces in completely edentulous individuals with mild-moderate OSA and compared it with conventional complete denture prosthesis. The null hypothesis stated that there was no difference in airway space dimensions among mild-moderate sleep apneic edentulous patients rehabilitated with complete dentures and implant-supported mandibular complete denture. 

## Materials and Method

Formal Ethical approval was obtained from the Institutional Ethics Committee of Sri Ramachandra Institute of Higher Education and Research (SRIHER, DU) (REFERENCE NO: CSP/19/NOV/81/420). Patients with completely edentulous arches who visited the Department of Prosthodontics were explained about the study and an informed consent was obtained. They were screened using the snoring, tiredness, observed apnea, high blood pressure, body mass index, age, neck circumference, and gender (STOP-Bang) questionnaire, which is specifically developed to meet the need for a reliable, concise, and easy-to-use screening tool [ 9 , 12 ]. It consists of eight dichotomous (yes/no) questions related to the clinical features of OSA. The patients were able to respond to most of the questions. When they were not aware of their behaviour during sleep, spouse or children answered those questions. For each question, answering “yes” scores 1, a “no” response scores 0, and the total score ranges from 0 to 8. Patients with a STOP-Bang score of 0 to 2 can be classified as low risk for moderate to severe OSA, scores in the midrange (3 or 4) as mode-rate risk for moderate to severe OSA, whereas those with a score of 5 to 8 can be classified as high risk for moderate to severe OSA (Table 1) [ 12 ]. Adopting 95% power in calculating sample size (G*Power 3.1.9.2) and regarding the study of Milosevic *et al*. [ 13 ], ten patients aged between 50-65 years, who were first time denture wearers with well-formed residual ridges in Class 1 relation and characterized into low-mode-rate risk category were included as study participants. Patients with history of any metabolic syndrome, surgery of tongue, palate or upper airways, skeletal l class III relationship, grossly resorbed residual alveolar ridges, and musculoskeletal disorders were excluded from the study. Standardised lateral cephalograms (X-ray device-CARESTR-EAM) were made for all the study participants at edentulous state and was considered baseline (L1). The participants were further screened using lateral cephalograms to eliminate nasal obstruction or pharyngeal tumours. 

**Table 1:** STOP-BANG Sleep Apnea Questionnaire

**Stop**		
Do you SNORE loudly( louder than talking or loud enough to be heard through closed doors)?	Yes	No
Do you often feel TIRED, fatigued, or sleepy during daytime?	Yes	No
Has anyone OBSERVED you stop breathing during your sleep?	Yes	No
Do you have or are you being treated for high blood PRESSURE?	Yes	No
**Bang**		
BMI more than 35kg/m?	Yes	No
AGE over 50 years old?	Yes	No
NECK circumference > 16 inches (40 cm)?	Yes	No
GENDER: Male?	Yes	No
**Total Score**		

High Risk of OSA: Yes 5-8

Intermediate risk of OSA: Yes 3-4

Low risk of OSA: Yes 0-2

*Chung, F., Abdullah, H., & Liao, P. (2016). STOP-Bang Questionnaire. Chest, 149(3), 631-638.

**Figure 1:**
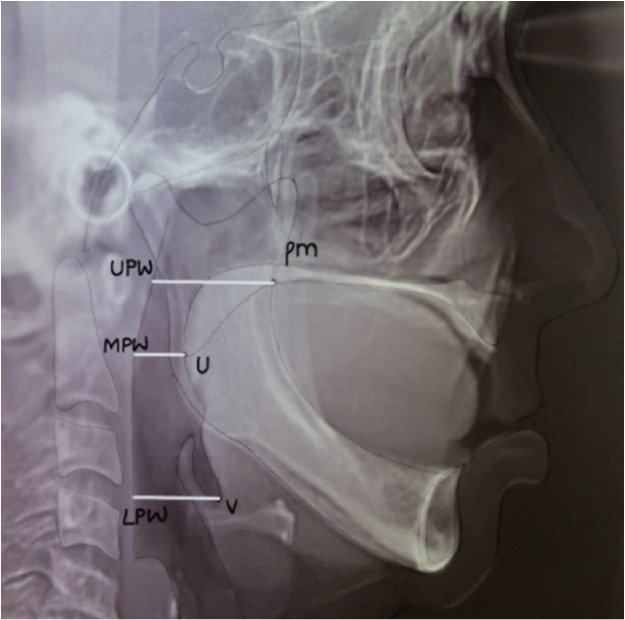
Cephalometric variables depicting Airway space

Cephalometric variables depicting the upper airway space (UAS), middle airway space (MAS), and lower airway space (LAS) were traced by the principal investigator following the standard procedure. UAS was defined as the distance between the pterygomaxillary and upper pharyngeal wall (pm‑UPW), MAS was the distance between the tip of the uvula and middle pharyngeal wall (U‑MPW), and LAS was the distance between the vallecula and the lower pharyngeal wall (V‑LPW) (Figure 1). The dimensions were measured in millimetres (mm) by both the investigators and tabulated. Subsequently, conventional complete dentures with bilateral balanced occlusion were fabricated by a single operator and inserted. Twenty-four-hour review was scheduled for post-operative adjustment. One week following the denture insertion, implant placement was planned in the canine region (B and D position) on the mandibular arch. The mandibular complete denture was duplicated using acrylic resin (Dental Products of India - clear heat cure) to function as a guide. A full thickness mucoperiosteal flap was elevated after administration of local anaesthesia. Sequential osteotomy was performed and dental implants of size 3.3*11.5mm (BIOLINE, India) were torque wrenched into the osteotomy sites, after which cover screws were placed. The flap was approximated using simple interrupted sutures. Study population was reviewed after twenty-four hours and denture bases were relieved to prevent discomfort.

Six months after complete denture insertion, study participants were recalled and lateral cephalogram (L2) was made to evaluate the change in airway dimensions brought about by complete dentures. Mandibular complete dentures were then converted to implant-supported denture using ball attachments (BIO-T1201, BIO-T1202) and corresponding metal caps (BIO-T3001). Occlusal prematurities were eliminated and the study participants were reviewed after 24 hours. Six months after implant-supported prosthesis, the airway spaces were re-evaluated with lateral cephalogram (L3). Although the effects of oral appliances on the airway are discernible within six to eight weeks, we evaluated after six months to enhance patient compliance with dentures. The collected data was analysed using SPSS software version 21. Repeated measures ANOVA was used to assess the mean difference between the values obtained at three different time intervals. In the above statistical tool, *p*< 0.05 was considered statistically significant. Intraclass correlation coefficient test was used to assess the inter-observer variability.

## Results

The Repeated measures ANOVA measured the mean difference between the airway dimensions (in mm) on lateral cephalograms made at three time intervals. On evaluating the UAS, MAS and LAS, the mean difference between L1- L2, L2 - L3 and L1- L3 was statistically significant with a *p*<0.05 as indicated in Tables 2, 3 and 4. Intraclass correlation coefficient test assessing the inter-observer variability revealed an average value of 0.828 indicating high similarity as seen in Table 5. Intragroup Post hoc analysis using Tukey HSD indicated that the difference between UAS and MAS, MAS and LAS, LAS and UAS was statistically significant at all three intervals as seen in Table 6. However, the difference in UAS was statistically more significant followed by LAS and MAS (α=.05). Intergroup Post hoc- analysis using Bonferroni test indicated that the difference in the UAS and MAS obtained at L2 and L3 was truly significant (α=.05) when compared with L1 as seen in Table 7. However, the differences in LAS were truly significant only at L3. 

**Table 2:** Assessment of upper airway space dimensions (UAS) in mm

Time intervals	Mean	Std. Deviation	N	*p* value
L1	2.60	.36	10	0.00
L2	2.80	.31	10	
L3	2.88	.30	10	

*p< .050 statistically significant

^a^L1 indicates edentulous state-baseline

^b^L2 indicates 6 months after complete denture rehabilitation

^c^L3 indicates 6 months after implant-supported mandibular complete rehabilitation

^d^N refers to number of samples

**Table 3:** Assessment of middle airway space dimensions (MAS) in mm

Time intervals	Mean	Std. Deviation	N	*p* value
L1	1.03	.20	10	0.00
L2	1.21	.12	10	
L3	1.40	.11	10	

* *p*<.050 statistically significant

^a^ L1 indicates edentulous state-baseline

^b^ L2 indicates 6 months after complete denture rehabilitation

^c^ L3 indicates 6 months after implant-supported mandibular complete rehabilitation

^d^ N refers to number of samples

**Table 4:** Assessment of lower airway space dimensions (LAS) in mm

Time Intervals	Mean	Std. Deviation	N	*p* value
L1	1.56	.35	10	0.00
L2	1.63	.21	10	
L3	1.78	.13	10	

* *p*<.050 statistically significant

^a^ L1 indicates edentulous state-baseline

^b^ L2 indicates 6 months after complete denture rehabilitation

^c^ L3 indicates 6 months after implant-supported mandibular complete rehabilitation

^d^ N refers to number of samples

**Table 5:** Intraclass correlation coefficient

	Intraclass Correlation	95% Confidence Interval		F Test with True Value 0	
		Lower Bound	Upper Bound	Value	Sig
Single Measures	.16	.06	.30	5.22	.00
Average Measures	.83	.63	.92	5.22	.00

**Table 6:** Post hoc Tukey HSD (Intragroup comparison)

Dependent Variable	Airway space comparison		Mean Difference (I-J) in mm	Sig.
L1	Upper	Middle	1.57*	.00
		Lower	1.04*	.00
	Middle	Upper	-1.57*	.00
		Lower	-.59*	.01
	Lower	Upper	-1.046*	.00
		Middle	.53*	.01
L2	Upper	Middle	1.58*	.00
		Lower	1.17*	.00
	Middle	Upper	-1.58*	.00
		Lower	-.41*	.00
	Lower	Upper	-1.17*	.00
		Middle	.41*	.00
L3	Upper	Middle	1.48*	.00
		Lower	1.10*	.00
	Middle	Upper	-1.48*	.00
		Lower	-.38*	.00
	Lower	Upper	-1.10*	.00
		Middle	.38*	.00

* *p*<. 050 statistically significant

^a^L1 indicates edentulous state-baseline

^b^L2 indicates 6 months after complete denture rehabilitation

^c^L3 indicates 6 months after implant-supported mandibular complete rehabilitation

**Table 7:** Post hoc Bonferroni test (Intergroup comparison)

Comparison between airway spaces at the three time intervals (L1, L2 and L3)			Mean Difference in mm	Sig.
UAS	L1	L2	-.20*	.00
		L3	-.29*	.00
	L2	L1	.20*	.00
		L3	-.09*	.00
	L3	L1	.29*	.00
		L2	.09*	.00
MAS	L1	L2	-.19*	.04
		L3	-.37*	.00
	L2	L1	.19*	.04
		L3	-.19*	.00
	L3	L1	.37*	.00
		L2	.19*	.00
LAS	L1	L2	-.07	1.00
		L3	-.23	.25
	L2	L1	.07	1.00
		L3	-.16*	.03
	L3	L1	.23	.25
		L2	.16*	.03

* *p*<.050 statistically significant

^a^ L1 indicates edentulous state-baseline

^b^ L2 indicates 6 months after complete denture rehabilitation

^c^ L3 indicates 6 months after implant-supported mandibular complete rehabilitation

^d^ UAS indicates upper airway space

^e^ MAS indicates middle airway space

^f^ LAS indicates lower airway space

## Discussion

Complete edentulism results in reduction of lower facial height, mandibular rotation, and impaired neuromuscular reflexes favouring upper airway collapse [ 2 - 4 , 11 , 14 - 15 ]. These changes along with abnormal tongue position predispose edentulous patients to OSA. Although an overnight polysomnography is the gold standard diagnostic test, it is time consuming, labour-intensive, expensive and requires the expertise of sleep medicine specialists [ 5 , 16 - 17 ]. Thus, a simple chairside screening tool is required to assist in identification. The STOP-Bang questionnaire has demonstrated high sensitivity in detection across various populations [ 9 ]. In this study, it has assisted in identifying patients with risk for OSA. 

Oral appliances were found to be more satisfactory for mild to moderate OSA patients and contraindicated in patients categorised with severe OSA [ 17 ]. Complete dentures have been used as an oral appliance for patients with mild-moderate OSA [ 18 - 19 ]. They improved airway space through positional changes in mandible, tongue, and soft tissue. However, the results of studies assessing the effectiveness of complete dentures on airway space done in the past were inconclusive [ 18 , 20 ]. This study aimed to evaluate the effect of complete dentures on the airway space for a period of six months and the improvement in airway space with implant-supported complete dentures was assessed in the same study population. Both male and female study participants had worn complete dentures fabricated using conventional method and routine materials thus providing a homogenous and representative sample [ 18 ]. Airway space dimensions were measured on lateral cephalograms by two investigators. The cephalometric method, despite being a static two-dimensional method to evaluate anatomical structures of the head and neck, has been useful in assessing airway space. Repeated measures ANOVA revealed a statistically significant increase in the UAS, MAS, and LAS dimensions in L2 made six months after insertion of complete denture when compared with dimensions in L1 made at completely edentulous state with a *p*< 0.05 (Tables 2-4). We speculate that the increase in airway dimensions could have occurred due to the positional changes of the mandible. Complete dentures help re-establish maxilla-mandibular relationship. This in turn helps in restoring the tongue to its normal position and prevents blocking of airways. Additionally, the tonicity and function of the surrounding soft tissues are restored, which helps reduce pharyngeal collapsibility [ 18 ]. The results of the present study were in correlation with the findings of Gao *et al*. [ 21 ], who confirmed the positive effect of complete dentures on enlarging upper airway. Erovigni *et al*. [ 22 ] also stated that complete dentures increased the diameters of the velopharynx, oropharynx in the sagittal plane as well as at the uvula level.

Although complete dentures are the first option for rehabilitating edentulous patients, the mandibular complete denture lacks sufficient retention and stability. This affects patient compliance in wearing complete dentures, deteriorating its positive influence on airway space. Implant-supported mandibular denture had evolved as a viable option for edentulous patients with improved retention and quality of life [ 11 ]. In this study, mandibular complete dentures of the participants were converted into an implant-supported denture. Lateral cephalograms (L3) obtained six months after implant-supported prosthesis assessed the effect of implant-supported mandibular complete denture on the airway space. The values obtained with L3 were compared with the values obtained at L1 and L2. Post Hoc Bonferroni test indicated that the differences in UAS, MAS and LAS were found to be statistically significant after wearing implant-supported mandibular complete dentures (L3) when compared with conventional complete dentures (L2) and at edentulous state (L1) at *p*< 0.05 (Table 7). This increase, when compared to conventional complete dentures, could have been due to better retention, stability, patient comfort, and compliance, which in turn contributes to improved muscle tone and function [ 23 ]. Post Hoc Tukey HSD assessed the difference in the airway dimensions at the three-time intervals between both complete denture and implant-supported mandibular denture. It indicated a significant increase in UAS followed by LAS and MAS with implant-supported mandibular complete denture (L3) (Table 6). Upper airway collapse has been found to be more common in patients with OSA [ 2 ]. Thus, the results of this study indicate that implant-supported dentures itself can act as an oral appliance to increase airway space in edentulous sleep apneic patients. Hoekema *et al*. [ 24 ] identified mandibular advancement device, which was retained with a single implant, to be effective in edentulous sleep apneic patients. Moreover, the quality of life and sleep characteristics substantially improved with prosthetic rehabilitation in completely edentulous patients who had mild-moderate OSA.

In this study, the edentulous population benefitted from both complete dentures and implant-supported mandibular complete dentures. Hence, the follow-up period evaluated the impact of both the modes of prosthetic rehabilitation on the airway space. No other study has assessed the effect of implant-supported mandibular complete dentures on the airway space dimension among edentulous patients. While this observational study included ten participants for evaluation, a randomized controlled trial with a larger sample size can help in validating the results. The cephalometric tool used in this study evaluated the antero-posterior diameter of the airway. Subsequently, a three-dimensional evaluation with a longer follow-up in edentulous individuals might assist in drawing conclusions that are more definitive.

## Conclusion

Within the limitations of this study, the following conclusions were drawn. Implant-supported mandibular complete dentures improved airway space dimensions when compared with conventional complete dentures in edentulous patients with mild-moderate OSA at the end of twelve months. Among the three airway dimensions, the increase in UAS was statistically significant when compared to MAS and LAS for both modes of rehabilitation indicating a positive influence of complete dentures on airway space. 

## References

[1] Marin JM, Carrizo SJ, Vicente E, Agusti AGN ( 2005). Long-term cardiovascular outcomes in men with obstructive sleep apnoea-hypopnoea with or without treatment with continuous positive airway pressure: an observational study. Lancet.

[2] Marshall NS, Wong KKH, Liu PY, Cullen SRJ, Knuiman MW, Grunstein RR ( 2008). Sleep apnea as an independent risk factor for all-cause mortality: the Busselton Health Study. Sleep.

[3] Okşayan R, Sökücü O, Uyar M, Topçuoğlu T ( 2015). Effects of edentulism in obstructive sleep apnea syndrome. Niger J Clin Pract.

[4] George CFP ( 2007). Sleep apnea, alertness, and motor vehicle crashes. Am J Respir Crit Care Med.

[5] Piskin B, Sentut F, Sevketbeyoglu H, Avsever H, Gunduz K, Kose M, et al ( 2010). Efficacy of a modified mandibular advancement device for a totally edentulous patient with severe obstructive sleep apnea. Sleep Breath.

[6] Piskin B, Sipahi C, Karakoc O, Atay A, Ciftci F, Tasci C, et al ( 2014). Effects of complete dentures on respiratory performance: spirometric evaluation. Gerodontology.

[7] Flemons WW, Remmers JE ( 1996). The diagnosis of sleep apnea: questionnaires and home studies. Sleep.

[8] Bayat M, Shariati M, Rakhshan V, Abbasi M, Fateh A, Sobouti F, et al ( 2017). Cephalometric risk factors of obstructive sleep apnea. Cranio.

[9] Amra B, Rahmati B, Soltaninejad F, Feizi A ( 2018). Screening questionnaires for obstructive sleep apnea: an updated systematic review. Oman Med J.

[10] Katoch S, Kumar M, Khosla A, Batra R, Kaur N ( 2016). Obstructive Sleep Apnea–Epidemiology, Consequencies and Prosthetic Rehabilitation. A Review. Dent J Advance Studies.

[11] Olaithe M, Bucks RS ( 2013). Executive dysfunction in OSA before and after treatment: a meta-analysis. Sleep.

[12] Chung F, Abdullah HR, Liao P ( 2016). STOP-Bang Questionnaire: A Practical Approach to Screen for Obstructive Sleep Apnea. Chest.

[13] Milosevic B, Sojic LT, Stancic I, Cerovic Z, Zvrko E ( 2016). Magnetic Resonance Imaging in Complete Denture Treated Edentulous Patients with Obstructive Sleep Apnea Syndrome-A Preliminary Study. J Oral Hyg Health.

[14] Gowda M, Sahoo NK, Guruprasada NK, Verma K ( 2016). Evaluation of Denture Wear on Upper Airway Dimensions and Oxygen Saturation in Completely Edentulous Patients. J Sleep Disord Ther.

[15] Gupta MA, Simpson FC ( 2015). Obstructive sleep apnea and psychiatric disorders: a systematic review. J Clin Sleep Med.

[16] Norman D, Loredo JS ( 2008). Obstructive sleep apnea in older adults. Clin Geriatr Med.

[17] Chen Q, Zou D, Feng H, Pan S ( 2017). Will wearing dentures affect edentulous patients’ breathing during sleep?. Sleep Breath.

[18] Gupta P, Thombare R, Pakhan AJ, Singhal S ( 2011). Cephalometric evaluation of the effect of complete dentures on retropharyngeal space and its effect on spirometric values in altered vertical dimension. ISRN Dent.

[19] Emami E, Nguyen PTH, Almeida FR, Feine JS, Karp I, Lavigne G, et al ( 2014). The effect of nocturnal wear of complete dentures on sleep and oral health related quality of life: study protocol for a randomized controlled trial. Trials.

[20] Mayson D, Neilan TG, Awad K, Malhotra A ( 2012). Obstructive sleep apnea in the elderly: extent of the problem and therapeutic options. Curr Cardiovasc Risk Rep.

[21] Gao XM, Zeng XL, Fu MK, Huang XZ ( 1999). Magnetic resonance imaging of the upper airway in obstructive sleep apnea before and after oral appliance therapy. Chin J Dent Res.

[22] Erovigni F, Graziano A, Ceruti P, Gassino G, De Lillo A, Carossa S ( 2005). Cephalometric evaluation of the upper airway in patients with complete dentures. Minerva Stomatol.

[23] Rosenberg R, Hirshkowitz M, Rapoport DM, Kryger M ( 2019). The role of home sleep testing for evaluation of patients with excessive daytime sleepiness: focus on obstructive sleep apnea and narcolepsy. Sleep Med.

[24] Hoekema A, de Vries F, Heydenrijk K, Stegenga B ( 2007). Implant-retained oral appliances: a novel treatment for edentulous patients with obstructive sleep apnea-hypopnea syndrome. Clin Oral Implants Res.

